# LBSizeCleav: improved support vector machine (SVM)-based prediction of Dicer cleavage sites using loop/bulge length

**DOI:** 10.1186/s12859-016-1353-6

**Published:** 2016-11-25

**Authors:** Yu Bao, Morihiro Hayashida, Tatsuya Akutsu

**Affiliations:** Laboratory of Mathematical Bioinformatics, Bioinformatics Center, Institute for Chemical Research, Kyoto University, Gokasho, Uji, Kyoto, 611-0011 Japan

**Keywords:** Dicer cleavage site, Support vector machine, Loop/bulge length

## Abstract

**Background:**

Dicer is necessary for the process of mature microRNA (miRNA) formation because the Dicer enzyme cleaves pre-miRNA correctly to generate miRNA with correct seed regions. Nonetheless, the mechanism underlying the selection of a Dicer cleavage site is still not fully understood. To date, several studies have been conducted to solve this problem, for example, a recent discovery indicates that the loop/bulge structure plays a central role in the selection of Dicer cleavage sites. In accordance with this breakthrough, a support vector machine (SVM)-based method called PHDCleav was developed to predict Dicer cleavage sites which outperforms other methods based on random forest and naive Bayes. PHDCleav, however, tests only whether a position in the shift window belongs to a loop/bulge structure.

**Result:**

In this paper, we used the length of loop/bulge structures (in addition to their presence or absence) to develop an improved method, LBSizeCleav, for predicting Dicer cleavage sites. To evaluate our method, we used 810 empirically validated sequences of human pre-miRNAs and performed fivefold cross-validation. In both 5p and 3p arms of pre-miRNAs, LBSizeCleav showed greater prediction accuracy than PHDCleav did. This result suggests that the length of loop/bulge structures is useful for prediction of Dicer cleavage sites.

**Conclusion:**

We developed a novel algorithm for feature space mapping based on the length of a loop/bulge for predicting Dicer cleavage sites. The better performance of our method indicates the usefulness of the length of loop/bulge structures for such predictions.

## Background

MicroRNAs (miRNAs) are a type of small RNAs with the length ∼22 nt, which perform the function of suppressing gene expression at the post-transcriptional level [1, 2]. Usually in vivo, a gene of a miRNA is transcribed to produce a long, primary miRNA (pri-miRNA) transcript, which is then processed into a ∼65-nt-long hairpin structure via cleavage by the Drosha (DGCR8) enzyme. Then, the resulting pre-miRNA is cleaved by another enzyme (termed Dicer) to generate a mature miRNA, which is ∼22 nt long [3]. Finally, the generated miRNA can be combined with an Argonaute protein to form the protein–miRNA complex, which can control various cellular progresses including development, cell death, and metabolism [4–6].

Dicer is a 1922-amino acid multidomain protein that belongs to the RNase III family. Dicer generally contains several domains including ATPase–helicase, DUF283 (a double-stranded-RNA–binding domain), PAZ (Piwi–Argonaute–Zwille) domain, two RNase III domains, and a dsRBD [7]. Dicer in various species may contain a different combination of these domains. Among these domains, the PAZ domain, RNase III domain, and dsRND are responsible for the function of substrate cleavage [8]. The cleavage occurs near the end of the terminal loop of pre-miRNA, introducing a cut into the hairpin.

Structural analysis of human Dicer revealed that the PAZ domain contains a 5p phosphate-binding pocket, which may be necessary for selection of a Dicer cleavage site [9]. There are also studies showing that the loop/bulge structure also determines the accuracy of cleavage activity [10, 11]. MacRae et al. reported that the 3p-terminal nucleotide of single-stranded RNA can affect Dicer binding [12]. In addition, Jin and Lee found that a single nucleotide polymorphism may be associated with miRNA regulation [13]. All these studies revealed that secondary structures of both the Dicer enzyme and cleavage substrates are essential for cleavage site determination.

With a better understanding of the features of selection of a Dicer cleavage site, researchers may be able to elucidate the mechanism of action of enzymes in the RNA III family as well as the processes of RNA inference. Thus, it is imperative to explore the factors affecting the accuracy of Dicer cleavage to gain better insights into the mechanism of Dicer cleavage. Recently, a support vector machine (SVM)-based method (PHDCleav) was developed to predict selection of Dicer cleavage sites [14]. They proposed feature space mappings from pre-miRNA nucleotide sequences on the basis of existence of a predicted loop/bulge structure. SVM is a state-of-the-art machine learning technology [15] that has been applied to various areas of pattern recognition in many biological fields such as protein classification [16–18], prediction of RNA secondary structure [19, 20], and drug–nondrug classification [21, 22].

In this paper, we made use of the length of loop/bulge structures and proposed a novel algorithm of feature space mapping called *LBSizeCleav*. To evaluate our method, we used 810 empirically valid sequences of pre-miRNAs from miRBase and performed fivefold cross-validation. In the 5p arm of pre-miRNAs, the proposed method attained higher accuracy (87.4%), whereas the best prediction result of PHDCleav corresponded to the accuracy of 84.0% (an extended binary pattern, a window of 14-nt size). In addition, in the 3p arm, the average prediction accuracy of our method reached 83.0%, whereas PHDCleav achieved up to 79.1% prediction accuracy. These results suggest that our method LBSizeCleav outperforms binary patterns of PHDCleav in predicting the position of Dicer cleavage sites. The better performance may in turn serve as the evidence that the features utilized by these two methods are necessary for Dicer cleavage selection.

## Methods

In this section, we provide a brief description of feature space mapping algorithms of PHDCleav using sequences and secondary structures and propose a novel algorithm for feature space mapping, LBSizeCleav, based on the length of a loop/bulge structure.

### Feature space mapping procedures of PHDCleav

Given a pre-miRNA sequence, a site between two successive nucleotides is mapped to a binary vector. In PHDCleav, a window is generated for each input sequence where for the positive pattern the center of the window is exactly located at the cleavage site of 5p (3p) arm and for the negative pattern the center of the window is located 6 nt away from the cleavage site of 5p(3p) arm. Since this is based on the assumption that a cleavage site can shift slightly (1-2 nt in biological experiments) but the chance is rare that Dicer cuts in the middle of mature miRNA, 6 nt could be changed under the principle that the center of the negative pattern is far enough from the real cleavage site. PHDCleav has shown that there is little affect to the accuracy of prediction even with the shifting of negative windows among the whole sequence of pre-miRNA.

A nucleotide in a window having the site at the center is converted to a four-dimensional vector as [1, 0, 0, 0], [0, 1, 0, 0], [0, 0, 1, 0], and [0, 0, 0, 1], for A, U, C, and G, respectively (see Table 1). Let *w* denote the size of the window, where *w* is a positive even number. Then, a 4*w*-dimensional vector is generated for the site.

**Table 1:** Binary patterns for nucleotides, A, U, C, G, and a loop/bulge structure, denoted by L, in PHDCleav [14] and LBSizeCleav with *k* ones based on sequences and predicted secondary structures

Mapping		Sequence	Structure
PHDCleav	A	[1,0,0,0]	[1,0,0,0]
	U	[0,1,0,0]	[0,1,0,0]
	C	[0,0,1,0]	[0,0,1,0]
	G	[0,0,0,1]	[0,0,0,1]
	L	−	[0,0,0,0]
Extended PHDCleav	A	−	[1,0,0,0,0]
	U		[0,1,0,0,0]
	C		[0,0,1,0,0]
	G		[0,0,0,1,0]
	L		[0,0,0,0,1]
LBSizeCleav	A	−	[1,0,0,0,0, … 0]
	U		[0,1,0,0,0, … 0]
	C		[0,0,1,0,0, … 0]
	G		[0,0,0,1,0, … 0]
	L		$[0, 0, 0, 0, 0, \dots, 0, \overbrace {1, \dots, 1}^{k}, 0, \dots ]$

In PHDCleav binary patterns each nucleotide is represented by a 4-dimensional vector, and in PHDCleav Extended patterns each nucleotide is represented by a 5-dimensional vector, while in LBSizeCleav the dimension of the vector is 3+*k*+*N*, in which *N* denotes the maximum number of length of loop/bulges among all the pre-miRNAs in the training dataset

There are many loops/bulges in the secondary structure of pre-miRNA where one arm contains extra nucleotides without counterparts in the other arm [23]. A recent study indicated that these loops/bulges play an important role in the selection of a Dicer cleavage site [10]. This observation suggests that the loop/bulge structure may be a feature that is useful for prediction of a Dicer cleavage site. The extended binary pattern of PHDCleav was developed on the basis of this assumption.

After obtaining the secondary structure from a given sequence by some prediction methods, in the extended binary pattern of PHDCleav, a nucleotide is converted to a five-dimensional vector as [1, 0, 0, 0, 0], [0, 1, 0, 0, 0], [0, 0, 1, 0, 0], [0, 0, 0, 1, 0], and [0, 0, 0, 0, 1], for A, U, C, G, and L, respectively, where L indicates that the corresponding nucleotide is predicted to be in a loop/bulge structure. Just as the nucleotides in the window, its complementary nucleotides are also converted to a feature vector. After that, the dimensionality of the vector is 10*w*.

### Feature space mapping of LBSizeCleav

It is reasonable to consider not only the position but also the length of loop/bulge structures. Therefore, we propose novel feature space mapping (LBSizeCleav) by introducing the length of a loop/bulge structure into the algorithm.

The binary pattern of LBSizeCleav is an extension of that of PHDCleav. Let *M* be the maximal length of loops and bulges of all the pre-miRNAs in a dataset, and suppose *L*_*l*_ indicates that the corresponding nucleotide is in a loop/bulge structure of length *l*. Here we introduce a new parameter named *k* into LBSizeCleav, which is a positive integer representing the effect of length of loops and bulges to the kernel computation. Then, we designate a nucleotide without any loop/bulge structure for *k* as a (*M*+*k*+3)-dimensional vector, namely, [1,0,0,0,…,0], [0,1,0,0,…,0], [0,0,1,0,0,…,0], [0,0,0,1,0,…,0] for A, U, C, and G, respectively (see Table 1). A nucleotide in a loop/bulge structure of length *l* is represented as [0,…,0,1,…,1,0,… ], where *k* ones appear from the (4+*l*)-th element to the (*k*+3+*l*)-th element. Thus, for window size *w*, a 2*w*(*M*+*k*+3)-dimensional vector is generated.

Let ***x***_1_ and ***x***_2_ be binary patterns of $L_{l_{1}}$ and $L_{l_{2}}$, respectively. If we use the inner product for kernel computation, then the inner product between the binary patterns is ***x***_1_·***x***_2_= max{*k*−|*l*_1_−*l*_2_|,0}. If we use the radial basis function (RBF) kernel, exp{−*γ*||***x***_1_−***x***_2_||^2^}= exp{−4*γ* min{(*l*_1_−*l*_2_)^2^,*k* ^2^}}, where *γ*>0. These values assume the maximum when *l*_1_=*l*_2_ and decrease according to the difference |*l*_1_−*l*_2_| and *k*, while k gets larger, the value changes of kernel function is more sensitive to the size of |*l*_1_−*l*_2_|, in this way by controlling the value k we could control the sensitivity of our method to length of loops and bulges. Since PHDCleav used radial basis function (RBF), we also selected RBF as our kernel function.

**Fig. 1:**
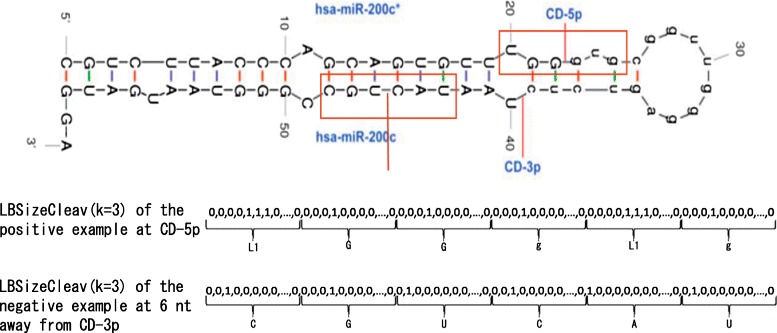
Illustration on the feature space mapping of LBSizeCleav. CD-5p and CD-3p denote cleavage sites in 5p and 3p arms, respectively, For two sites of CD-5p and six nucleotides far from CD-3p, the feature vectors of LBSizeCleav with *k*=3 and *w*=6 are shown, the *red rectangles* represent the window of the positive pattern of CD-5p and the window of the negative pattern of CD-3p

Figure 1 illustrates the feature space mapping of LBSizeCleav for the pre-miRNA of the miRBase ID hsa-miR-200c with a predicted secondary structure, where nucleotides in the region removed by Dicer are shown as lowercase letters. CD-5p and CD-3p denote cleavage sites in 5p and 3p arms, respectively. Sequences in the red rectangles denote sequences used to generate feature vectors representing 5p and 3p arms, which are selected by the principle that the cleavage site is located at the center of the sequence. Here, we generate the feature vector of LBSizeCleav at *k*=3 and *w*=6 for the site CD-5p and for the site 6 nt away from CD-5p. The nucleotides in the window in the 5p arm are UGGgug, and loop/bulge structures are detected at two positions. As a result, *L*_1_ *GGg* *L*_1_g is converted to the 6(*M*+6)-dimensional binary vector, where loop/bulge structures *L*_*l*_ are inserted. For the 3p arm, CGUCAU is converted in accordance with Table 1.

## Results

We retrieved 810 empirically validated sequences of pre-miRNAs from miRBase (version 21) [24], where cleavage sites CD-5p and CD-3p are both defined for each pre-miRNA. The pre-miRNAs were selected under the principle that both the cleavage sites of CD-5p and CD-3p are experimentally validated. (i.e. only precursors with cleavage sites at both CD-5p and CD-3p are selected, we made this choice to let our dataset be generated the in same way as dataset of PHDCleav) All the pre-miRNAs are selected from human premiRNAs.

We used these cleavage sites as positive examples using windows of size 8,10,12,14 nt, where each window was selected so that a cleavage site is located at the center of the window, and we generated negative examples on the same sequence so that both centers of the positive and negative examples were 6 nt away from each other, as in the previous study [14]. This approach is based on the assumption that for most pre-miRNAs, the Dicer cleavage site is seldom selected at the center of the hairpin structure. In PHDCleav, two secondary structure predictors, quikfold [25] and RNAFold from ViennaRNA [26] were used, hence, we used both the RNAFold from ViennaRNA. and the quikfold server (version 3.0, http://mfold.rna.albany.edu/?q=DINAMelt/Quickfold) for prediction of RNA secondary structures. The results were given in Tables 2 and 3. Because in PHDCleav, the accuracy of prediction by nucleotide composition was worse than that by binary patterns, we compared our method with the binary patterns of PHDCleav. We used the libSVM 3.18 package [27] with the RBF kernel to utilize SVM because the RBF kernel was used in PHDCleav.

**Table 2:** Results on average specificity, sensitivity, accuracy, and MCC for both 5p and 3p arms by five-fold cross-validation using PHDCleav and LBSizeCleav (*k*=1,⋯,5) with window sizes 8,10,12,14 based on sequences and secondary structures predicted by quikfold server

Method	Window size	5p arm				3p arm			
		Sn	Sp	Ac	MCC	Sn	Sp	Ac	MCC
PHDCleav (sequence)	8	0.602	0.503	0.552	0.105	0.662	0.625	0.644	0.287
	10	0.541	0.573	0.557	0.115	0.661	0.642	0.652	0.303
	12	0.560	0.555	0.557	0.115	0.660	0.656	0.658	0.316
	14	0.539	0.572	0.555	0.111	0.654	0.702	0.678	0.356
PHDCleav (structure)	8	0.753	0.814	0.784	0.568	0.670	0.661	0.665	0.330
	10	0.784	0.827	0.806	0.612	0.702	0.719	0.710	0.421
	12	0.790	0.842	0.816	0.633	0.739	0.764	0.752	0.503
	14	0.799	0.857	0.828	0.657	0.779	0.783	0.781	0.562
Extended PHDCleav	8	0.750	0.798	0.774	0.548	0.652	0.716	0.684	0.369
	10	0.779	0.827	0.803	0.607	0.674	0.783	0.729	0.460
	12	0.809	0.845	0.827	0.654	0.714	0.790	0.752	0.506
	14	0.813	0.868	0.840	0.682	**0.781**	0.801	0.791	0.582
LBSizeCleav (*k*=1)	8	0.668	0.924	0.796	0.612	0.630	0.684	0.657	0.315
	10	0.709	0.947	0.828	0.675	0.651	0.776	0.713	0.430
	12	0.774	0.945	0.859	0.730	0.686	0.847	0.766	0.540
	14	0.808	0.933	0.871	0.747	0.758	0.874	0.816	0.637
LBSizeCleav (*k*=2)	8	0.662	**0.954**	0.808	0.645	0.626	0.723	0.674	0.351
	10	0.725	0.946	0.835	0.688	0.642	0.806	0.724	0.455
	12	0.784	0.938	0.861	0.731	0.665	0.882	0.773	0.560
	14	0.820	0.925	0.872	**0.749**	0.734	0.916	0.825	0.661
LBSizeCleav (*k*=3)	8	0.692	0.949	0.821	0.664	0.619	0.735	0.677	0.356
	10	0.752	0.941	0.846	0.706	0.618	0.822	0.720	0.450
	12	0.803	0.932	0.867	0.741	0.635	0.914	0.774	0.571
	14	0.825	0.912	0.869	0.740	0.719	**0.942**	**0.830**	**0.678**
LBSizeCleav (*k*=4)	8	0.695	0.949	0.822	0.667	0.614	0.736	0.675	0.353
	10	0.767	0.938	0.853	0.716	0.621	0.835	0.728	0.467
	12	0.815	0.927	0.871	0.747	0.639	0.912	0.776	0.573
	14	0.835	0.909	0.872	0.746	0.723	0.924	0.823	0.660
LBSizeCleav (*k*=5)	8	0.700	0.947	0.824	0.668	0.594	0.771	0.682	0.371
	10	0.772	0.936	0.854	0.717	0.578	0.862	0.720	0.459
	12	0.821	0.924	0.872	**0.749**	0.634	0.921	0.777	0.579
	14	**0.838**	0.909	**0.874**	**0.749**	0.724	0.932	0.828	0.671

Sn, Sp, Ac, and MCC denote sensitivity, specificity, accuracy, and Matthews correlation coefficient, respectively

**Table 3:** Results on average specificity, sensitivity, accuracy, and MCC for both 5p and 3p arms by five-fold cross-validation using PHDCleav and LBSizeCleav (*k*=1,⋯,5) with window sizes 8,10,12,14 based on secondary structures predicted by RNAFold

Method	Window size	5p arm				3p arm			
		Sn	Sp	Ac	MCC	Sn	Sp	Ac	MCC
Extended PHDCleav	8	0.746	0.744	0.745	0.490	0.772	0.750	0.761	0.522
	10	0.792	0.783	0.787	0.575	0.779	0.800	0.790	0.580
	12	0.798	0.799	0.798	0.597	0.785	0.830	0.808	0.616
	14	0.778	0.813	0.795	0.591	0.805	**0.853**	0.829	0.659
LBSizeCleav (*k*=1)	8	0.739	0.805	0.772	0.545	0.785	0.790	0.788	0.576
	10	0.798	0.820	0.809	0.618	0.795	0.815	0.805	0.610
	12	0.792	0.815	0.803	0.607	0.822	0.840	0.831	0.662
	14	0.815	**0.822**	**0.819**	**0.638**	0.851	0.852	**0.851**	**0.703**
LBSizeCleav (*k*=2)	8	0.753	0.788	0.771	0.542	0.792	0.788	0.790	0.580
	10	0.816	0.795	0.806	0.612	0.811	0.794	0.803	0.606
	12	0.836	0.784	0.810	0.621	0.814	0.803	0.808	0.617
	14	0.845	0.769	0.807	0.616	0.867	0.800	0.834	0.669
LBSizeCleav (*k*=3)	8	0.751	0.794	0.773	0.546	0.784	0.797	0.790	0.581
	10	0.808	0.808	0.808	0.615	0.795	0.813	0.804	0.608
	12	0.822	0.800	0.811	0.623	0.808	0.835	0.821	0.643
	14	0.816	0.803	0.809	0.619	0.853	0.838	0.846	0.692
LBSizeCleav (*k*=4)	8	0.764	0.772	0.768	0.536	0.809	0.772	0.790	0.581
	10	0.824	0.762	0.793	0.587	0.824	0.766	0.795	0.590
	12	0.841	0.737	0.789	0.581	0.842	0.756	0.799	0.600
	14	0.871	0.678	0.774	0.559	0.898	0.697	0.797	0.607
LBSizeCleav (*k*=5)	8	0.782	0.747	0.764	0.529	0.822	0.744	0.783	0.568
	10	0.836	0.732	0.784	0.572	0.829	0.726	0.777	0.558
	12	0.867	0.699	0.783	0.574	0.864	0.682	0.773	0.556
	14	**0.899**	0.626	0.763	0.546	**0.917**	0.619	0.768	0.562

Sn, Sp, Ac, and MCC denote sensitivity, specificity, accuracy, and Matthews correlation coefficient, respectively

The performance of prediction methods was assessed by means of sensitivity, specificity, accuracy, and the Matthews correlation coefficient (MCC), defined as follows:

1 $$\begin{array}{@{}rcl@{}} sensitivity\! &=&\! \frac{TP}{TP + FN}, \end{array} $$

2 $$\begin{array}{@{}rcl@{}} specificity\! &=&\! \frac{TN}{TN+FP}, \end{array} $$

3 $$\begin{array}{@{}rcl@{}} accuracy\! &=&\! \frac{TP+TN}{TP+FP+TN+FN}, \end{array} $$

4 $$\begin{array}{@{}rcl@{}} MCC\! &=&\!\! \frac{TP \cdot TN-FP \cdot FN}{\sqrt{(TP \,+\, FP)(TP \,+\, FN)(TN \,+\, FP)(TN \!\,+\,\! FN)}}, \end{array} $$

where *T* *P*,*T* *N*,*F* *P*,*F* *N* denote the number of true positive, true negative, false positive, and false negative results, respectively.

We performed fivefold cross-validation, and used the average sensitivity, specificity, accuracy, and MCC. We examined size *w* of a window from 8 to 14 and the number *k* of ones in LBSizeCleav from 1 to 5 for 5p and 3p arms of pre-miRNAs. Table 2 shows the results of PHDCleav and LBSizeCleav (*k*=1,⋯,5) based on sequences and secondary structures predicted by the quikfold server. In terms of prediction performance in the 5p arm of pre-miRNA, the best result of PHDCleav corresponded to the accuracy of 84.0%, whereas LBSizeCleav at *k*=5 achieved the accuracy of 87.4%. In addition, the values of prediction accuracy of LBSizeCleav at *w*=12,14 were higher than those of PHDCleav. As for prediction performance in the 3p arm of pre-miRNA, the best result of PHDCleav corresponded to the accuracy of 79.1%, whereas LBSizeCleav achieved the accuracy of 83.0%.

Table 3 shows the results of PHDCleav and LBSizeCleav (*k*=1,⋯,5) based on sequences and secondary structures predicted by the RNAFold. In terms of prediction performance in the 5p arm of pre-miRNA, the best result of PHDCleav corresponded to the accuracy of 81.3%, whereas LBSizeCleav at *k*=1 achieved the accuracy of 82.2%. As for prediction performance in the 3p arm of pre-miRNA, the best result of PHDCleav corresponded to the accuracy of 82.9%, whereas LBSizeCleav achieved the accuracy of 85.1%.

**Fig. 2:**
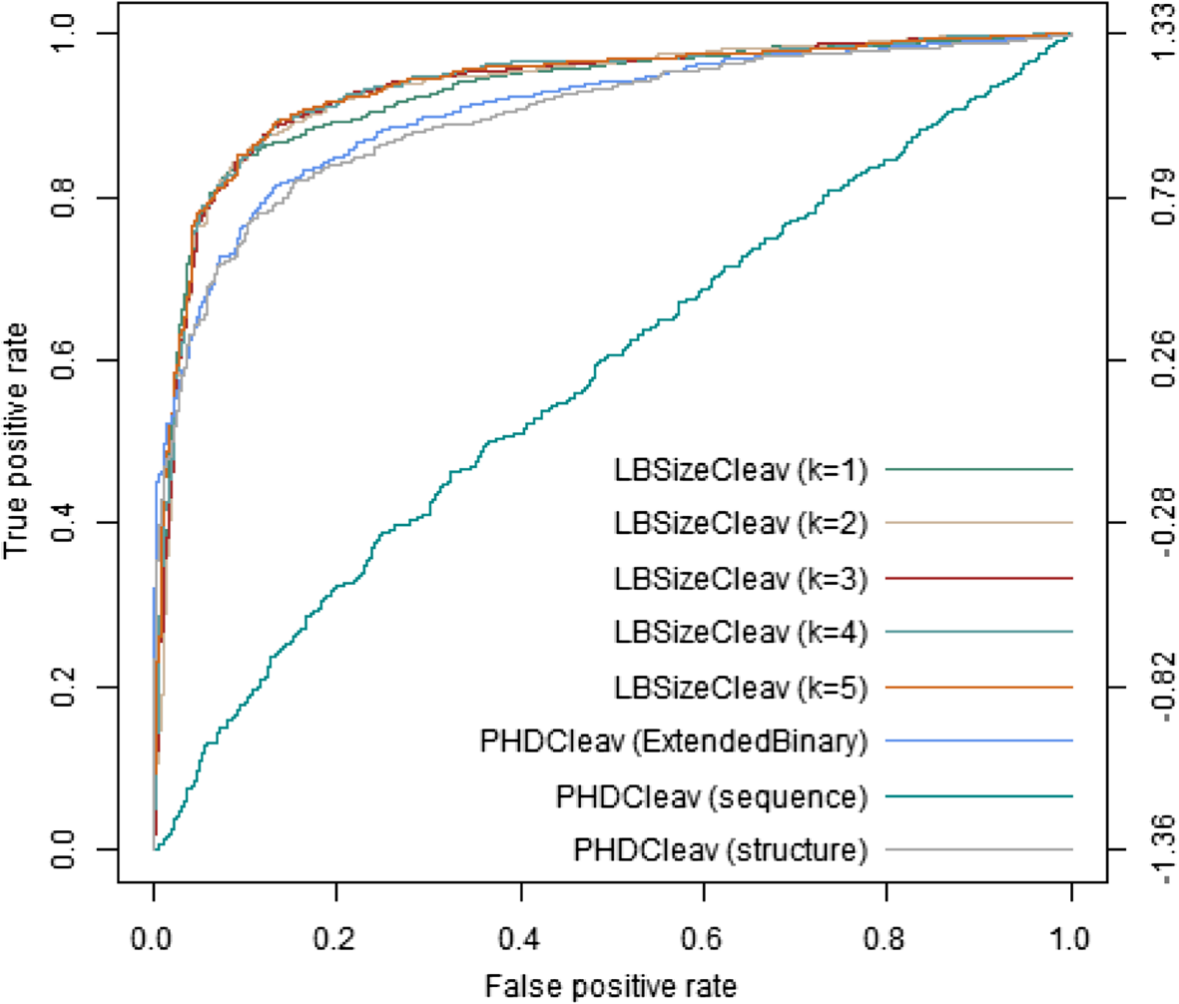
Results on ROC *curves* by LBSizeCleav and PHDCleav with window size *w*=14 for 5p arm. From the figure we could see that the ROC *curve* of LBSizeCleav from *k*=1 to *k*=5 is significantly better than binary Pattern and extended binary pattern of PHDCleav for both 5p and 3p arms

**Fig. 3:**
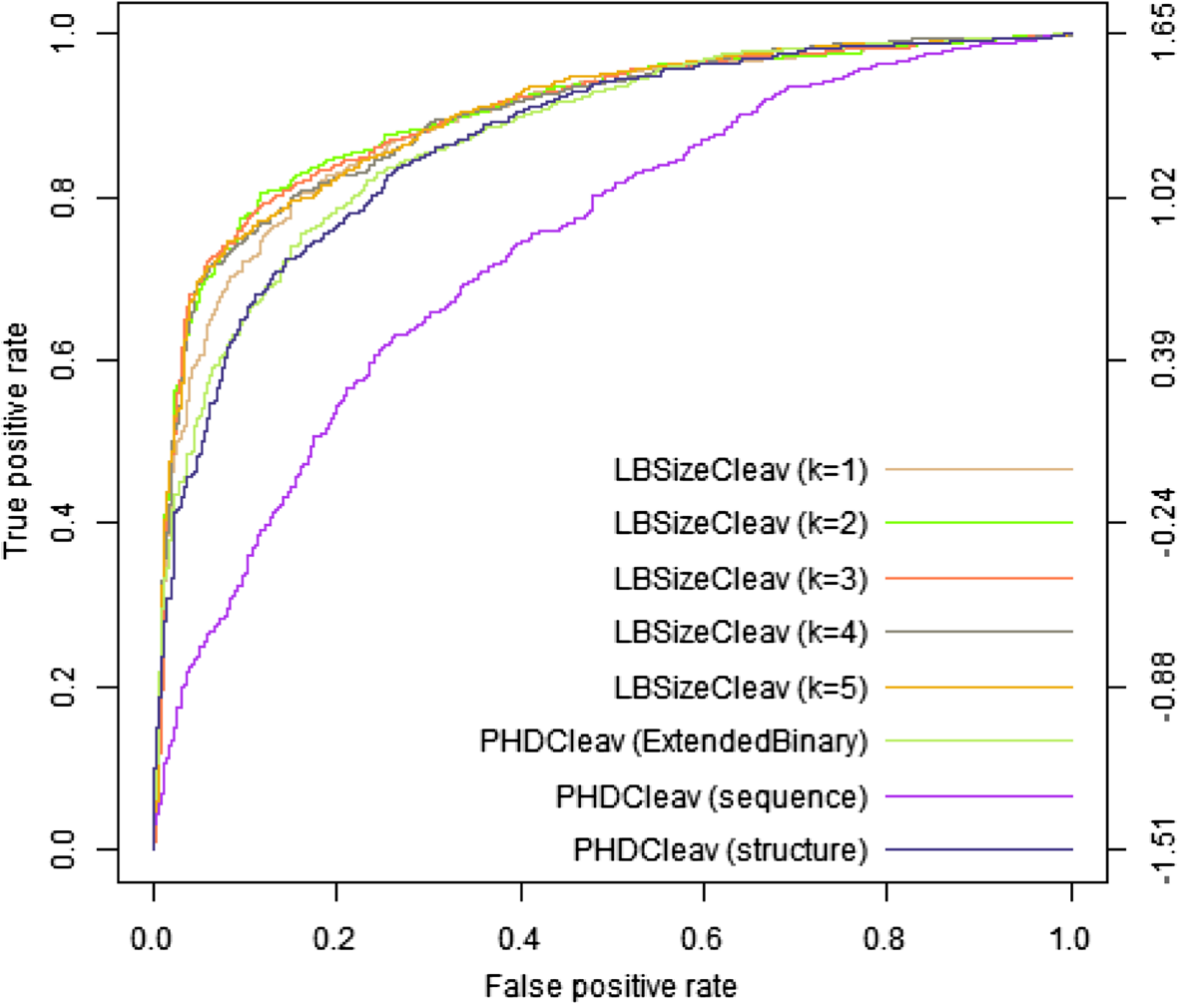
Results on ROC *curves* by LBSizeCleav and PHDCleav with window size *w*=14 for 3p arm. From the figure we could see that the ROC *curve* of LBSizeCleav from *k*=1 to *k*=5 is significantly better than binary Pattern and extended binary pattern of PHDCleav for both 5p and 3p arms

To better evaluate the performance we also calculated the variance of each prediction result in Table 4. Figures 2 and 3 show the results of LBSizeCleav and PHDCleav on receiver-operator characteristic (ROC) curves at window size *w*=14 in 5p and 3p arms. Judging by the performance evaluation, our newly developed method outperformed the binary patterns of PHDCleav; this finding was suggestive of efficiency of the feature representing the length of loop/bulge structures.

**Table 4:** Variances of specificity, sensitivity, accuracy, and MCC for both 5p and 3p arms by five-fold cross-validation using PHDCleav and LBSizeCleav (*k*=1,⋯,5) with window sizes 8,10,12,14 based on sequences and secondary structures predicted by quikfold server

feature extraction method	Window size	CD-5p				CD-3p			
		Sn	Sp	Ac	Mc	Sn	Sp	Ac	Mc
PHDCleav (sequence)	8	0.0137	0.0008	0.0074	0.0036	0.0072	0.0015	0.0094	0.0066
	10	0.0184	0.0004	0.0111	0.0018	0.0044	0.0005	0.0072	0.0024
	12	0.0208	0.0001	0.0223	0.0003	0.0078	0.0011	0.0031	0.0046
	14	0.0293	0.0009	0.0174	0.0037	0.0065	0.0007	0.0048	0.0029
PHDCleav (structure)	8	0.0042	0.0039	0.0067	0.0155	0.0187	0.0013	0.0091	0.0062
	10	0.0026	0.0043	0.0088	0.0177	0.0100	0.0014	0.0050	0.0059
	12	0.0042	0.0027	0.0034	0.0109	0.0051	0.0011	0.0024	0.0045
	14	0.0047	0.0031	0.0034	0.0125	0.0039	0.0012	0.0014	0.0047
Extended PHDCleav	8	0.0029	0.0032	0.0063	0.0128	0.0123	0.0025	0.0043	0.0103
	10	0.0030	0.0038	0.0061	0.0154	0.0064	0.0019	0.0016	0.0075
	12	0.0040	0.0033	0.0050	0.0136	0.0054	0.0015	0.0011	0.0059
	14	0.0059	0.0027	0.0016	0.0108	0.0032	0.0013	0.0010	0.0052
LBSizeCleav (*k*=1)	8	0.0030	0.0025	0.0044	0.0115	0.0100	0.0004	0.0074	0.0019
	10	0.0022	0.0015	0.0013	0.0064	0.0078	0.0011	0.0015	0.0042
	12	0.0050	0.0024	0.0010	0.0090	0.0077	0.0015	0.0002	0.0051
	14	0.0075	0.0035	0.0010	0.0132	0.0036	0.0007	0.0002	0.0026
LBSizeCleav (*k*=2)	8	0.0042	0.0018	0.0008	0.0066	0.0053	0.0010	0.0036	0.0041
	10	0.0038	0.0020	0.0009	0.0076	0.0034	0.0010	0.0010	0.0039
	12	0.0051	0.0029	0.0016	0.0115	0.0042	0.0017	0.0008	0.0063
	14	0.0051	0.0028	0.0012	0.0107	0.0043	0.0008	0.0002	0.0024
LBSizeCleav (*k*=3)	8	0.0025	0.0013	0.0008	0.0050	0.0070	0.0010	0.0021	0.0042
	10	0.0039	0.0022	0.0012	0.0086	0.0064	0.0013	0.0006	0.0048
	12	0.0063	0.0031	0.0012	0.0119	0.0039	0.0015	0.0005	0.0055
	14	0.0060	0.0033	0.0015	0.0130	0.0073	0.0016	0.0003	0.0046
LBSizeCleav (*k*=4)	8	0.0029	0.0016	0.0009	0.0064	0.0066	0.0020	0.0030	0.0078
	10	0.0046	0.0025	0.0011	0.0095	0.0071	0.0014	0.0006	0.0049
	12	0.0061	0.0032	0.0014	0.0124	0.0033	0.0011	0.0007	0.0041
	14	0.0051	0.0030	0.0015	0.0120	0.0088	0.0025	0.0002	0.0082
LBSizeCleav (*k*=5)	8	0.0029	0.0017	0.0009	0.0066	0.0062	0.0021	0.0031	0.0082
	10	0.0055	0.0029	0.0013	0.0113	0.0032	0.0011	0.0005	0.0042
	12	0.0051	0.0029	0.0015	0.0114	0.0029	0.0011	0.0009	0.0044
	14	0.0047	0.0029	0.0016	0.0116	0.0076	0.0023	0.0002	0.0076

Sn, Sp, Ac, and MCC denote sensitivity, specificity, accuracy, and Matthews correlation coefficient, respectively

**Fig. 4:**
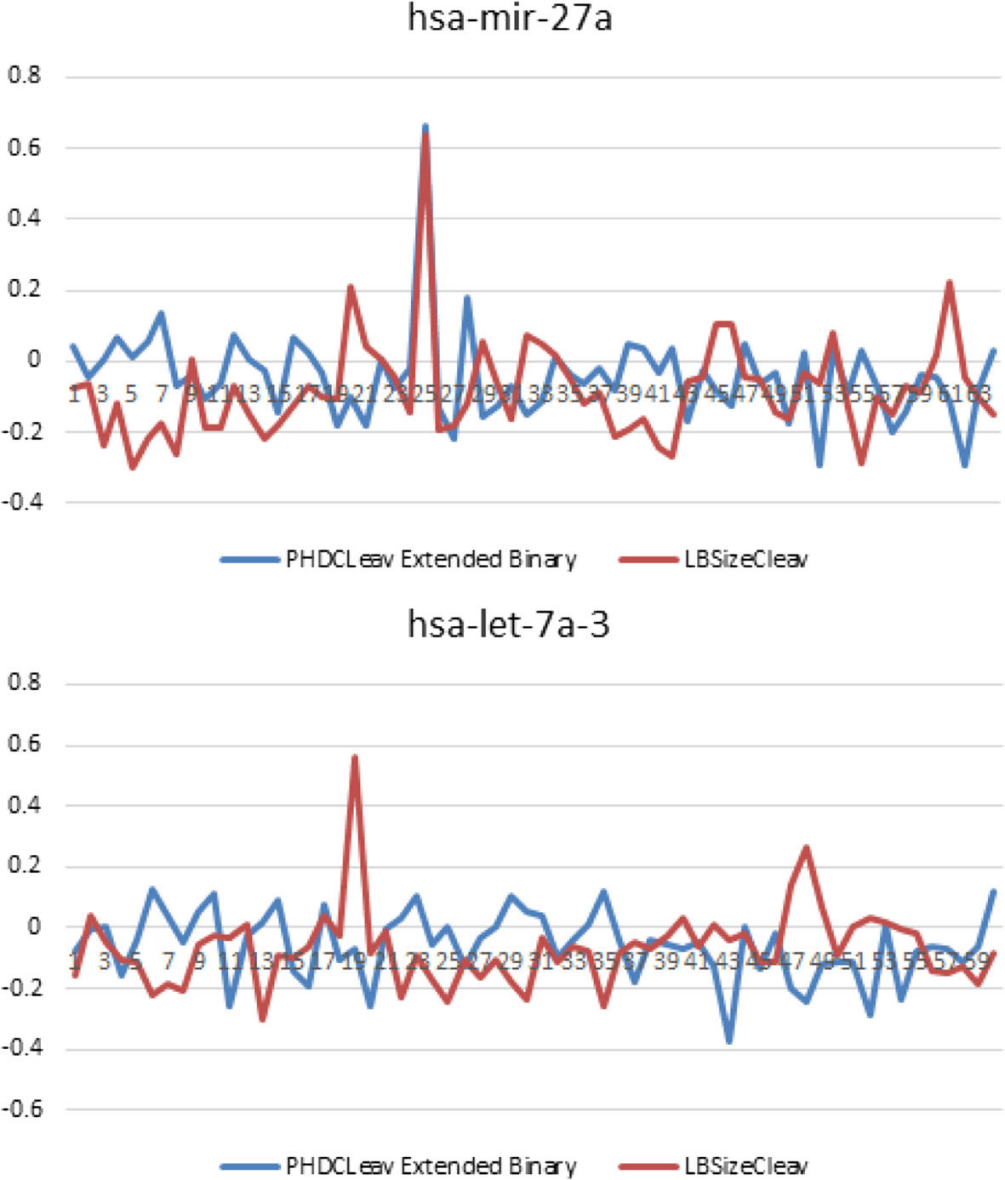
Regression analysis examples of LBSizeCleav(*k*=5) compared with PHDCleav extended binary

Since in our results, LBSizeCleav with parameters of *w*=14,*k*=5 outperforms the others, we selected these parameters as our parameters for prediction model. For an input sequence, we created a shift window of size 14 nt shifting from the 5p arm to the 3p arm. For each shift window we performed an SVM regression analysis using our model. Here we randomly selected 2 precursors from the dataset and showed the score of the extended binary pattern of PHDCleav and LBSizeCleav with *k*=5. From the result we could see that although both tools have predicted the cleavage site correctly, LBSizeCleav predicted more true negatives than extended binary pattern of PHDCleav, which indicates a better performance in identifying negative patterns of LBSizeCleav (see in Fig. 4).

**Fig. 5:**
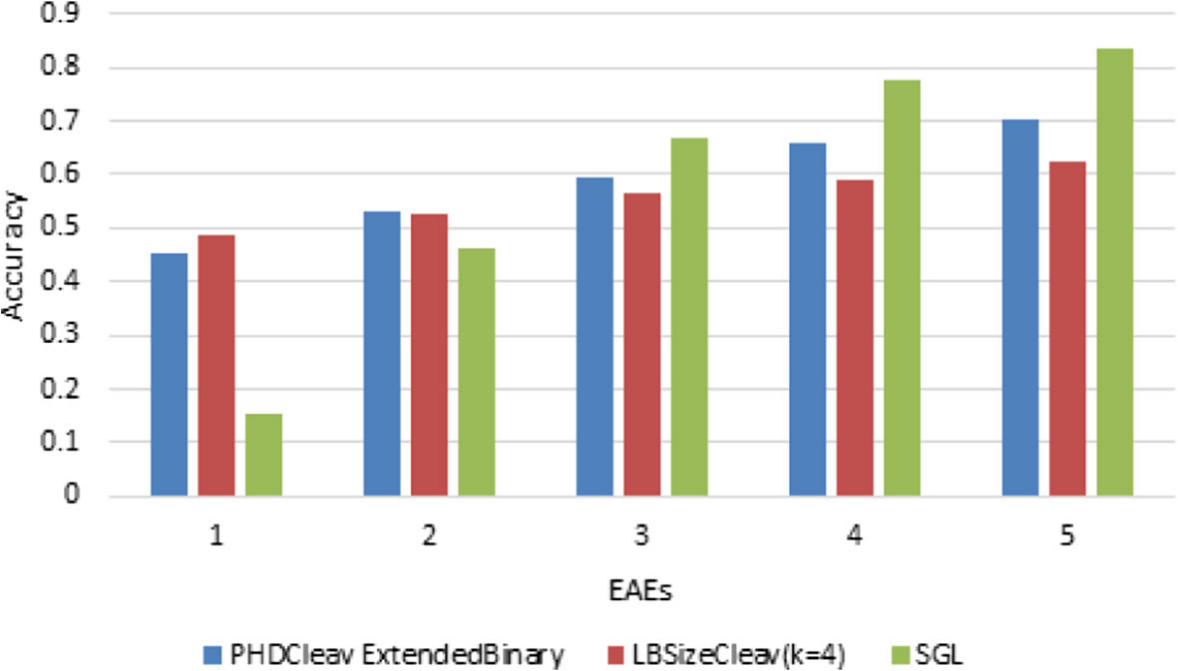
Result on accuracy of LBSizeCleav(*k*=5) compared with PHDCleav extended binary and SGL of prediction in CD-5p

We also compared the performance of our tools with another state-of-art method, a recent published paper introduced an easy way named SGL (Simple Geometric Locater) to calculate the cleavage site of miRNA which outperforms other methods. We generated a benchmark to compare our method as well as PHDCleav with SGL of prediction in CD-5p, which result is shown in Fig. 5. In this benchmark we selected the threshold (0.0) of LBSizeCleav as well as PHDCleav and calculated the EAEs(End Absolute Error, the absolute error of the predicted minus the true position for a specific duplex end) from the true cleavage site and compared it with the SGL method. From the result we could see that although at high EAEs PHDCleav outperforms LBSizeCleav, LBSizeCleav outperforms both PHDCleav and SGL at EAE 1, which indicates that LBSizeCleav predicted less false positives than PHDCleav.

## Discussion

**Fig. 6:**
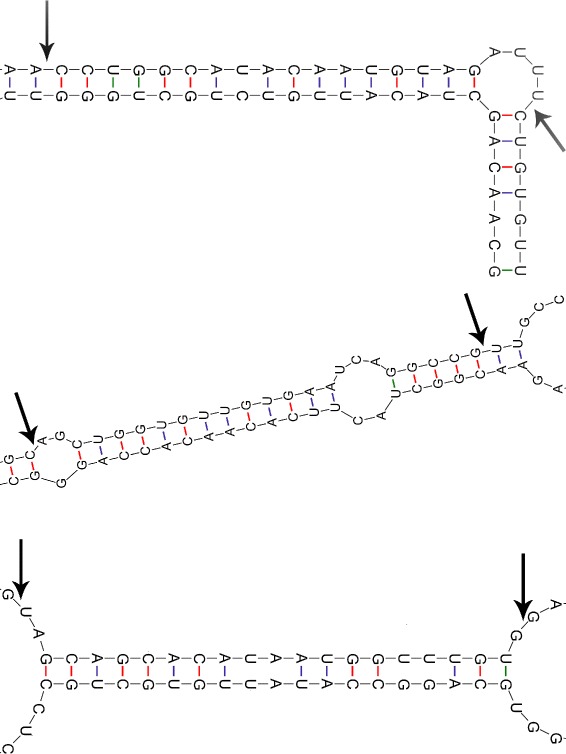
Secondary structures of hsa-mir-221, hsa-mir-138-1, hsr-mir-15a predicted by quikfold server. The *black arrow* means the cleavage site validated by biological experiments

There were several pre-miRNAs, such as pre-mir221, pre-mir138-1, and pre-mir-15a, that were identified by LBSizeCleav but were not identified by PHDCleav in the prediction results from the 5p arm of a pre-miRNA with a shift window of 14 nt (see Fig. 6). By comparing these three pre-miRNAs, we found that all of them contain a part of loop/bulge structures that is more than 1 nt long in their mature parts. This result indicates that the length of a loop/bulge structure is an important determinant of a cleavage site. Careful analysis revealed that pre-mir221 and pre-mir138-1 contain their loop/bulge structures in their bulge parts, whereas pre-mir-15a has its loop/bulge structure in its loop part, proving that both loop and bulge structures can affect the cleavage site selection. To further evaluate the effect of the length of a loop/bulge structure in affecting the cleavage site selection we calculated the number of pre-miRNAs which LBSizeCleav identified successfully while PHDCleav failed to identify and vice versa (see Table 5). From the result we could see that for the positive patterns our method performs almost the same as PHDCleav, but for negative patterns our method shows a significant improvement in accuracy. This result indicates that our method has a better resolution in identifying negative patterns.

**Table 5:** Number of patterns predicted only by LBSizeCleav(*k*=1,4)/PHDCleav(extended binary) using secondary structure predicted by quikfold

		5’-arm	3’-arm
Positive	Only predicted by LBSizeCleav (*k*=1) compared with PHDCleav (extended binary)	39	39
	Only predicted by PHDCleav (extended binary) compared with LBSizeCleav (*k*=1)	57	38
Negative	Only predicted by LBSizeCleav (*k*=1) compared with PHDCleav (extended binary)	82	65
	Only predicted by PHDCleav (extended binary) compared with LBSizeCleav (*k*=1)	23	12
Positive	Only predicted by LBSizeCleav (*k*=4) compared with PHDCleav(extended binary)	39	39
	Only predicted by PHDCleav compared with LBSizeCleav (*k*=4)	57	38
Negative	Only predicted by LBSizeCleav (*k*=4) compared with PHDCleav(extended binary)	82	65
	Only predicted by PHDCleav (extended binary) compared with LBSizeCleav (*k*=4)	23	12

## Conclusions

In this study, we proposed a novel method—LBSizeCleav—for prediction of Dicer cleavage sites. By integrating information on the length of a loop/bulge structure of a pre-miRNA (as predicted by the quikfold server), we developed novel feature space mapping. We performed fivefold cross-validation for validated pre-miRNA sequences from miRBase. In both 5p and 3p arms, the proposed method showed better performance than did the binary patterns of PHDCleav. This study shows a new way of feature evaluation; moreover, the better performance of our method points to the effectiveness of analysis of loop/bulge length at detecting Dicer cleavage sites.

## Abbreviations

DGCR8: DiGeorge syndrome chromosomal region 8
dsRND: Double-stranded RNA-binding domain
FN: false negative
FP: False positive
miRNA: MicroRNA
PAZ: PIWI, AGO, and Zwille domain
RISC: RNA-induced silencing complex
SVM: Support Vector Machine
TN: True negative
TP: True positive
